# Study on UF PES Membranes Spray-Coated with Polymerizable Bicontinuous Microemulsion Materials for Low-Fouling Behavior

**DOI:** 10.3390/membranes13120893

**Published:** 2023-11-29

**Authors:** Sneha De, Jonathan Heer, Suwetha Sankar, Fabian Geiger, Ephraim Gukelberger, Francesco Galiano, Raffaella Mancuso, Bartolo Gabriele, Alberto Figoli, Jan Hoinkis

**Affiliations:** 1 Center of Applied Research (CAR), Karlsruhe University of Applied Sciences (HKA), Moltkestrasse 30, 76133 Karlsruhe, Germany; 2 Laboratory of Industrial and Synthetic Organic Chemistry (LISOC), Department of Chemistry and Chemical Technology (CTC), University of Calabria, Via P. Bucci 12/C, 87036 Rende, CS, Italy; raffaella.mancuso@unical.it (R.M.);; 3 Institute on Membrane Technology (ITM-CNR), Via P. Bucci 17c, 87036 Rende, CS, Italy

**Keywords:** membrane fouling, fouling propensity, low-fouling layer, PBM-coated membranes, spray coating, critical flux

## Abstract

The low-fouling propensity of commercially available polyethersulfone (PES) membranes was studied after modification of the membrane surface via coating with polymerizable bicontinuous microemulsion (PBM) materials. The PBM coating was polymerized within 1 min using ultraviolet (UV) light exposure. It was detected on the PES membrane surface via attenuated total reflectance-Fourier transform infrared (ATR-FTIR) spectroscopy. The PBM coating led to an average 10% increase in the hydrophilicity of the PES membrane surface and an increase in total organic content (TOC) removal by more than 15%. Flux-step tests were conducted with model foulant comprising 100 mg L^−1^ humic acid (HA) solution to detect the onset of critical fouling, characterized by a rapid and substantial increase in TMP, and to compare the fouling propensity of commercially available PES membranes with PBM-coated membranes. The critical flux was found to be about 40% higher for PBM spray-coated membrane and 20% lower for PBM casting-coated membrane than the commercial PES membrane. This demonstrates the performance advantages of the thin PBM layer spray-coated on PES membrane compared to the thick casting-coated PBM layer. The study showcases the potential of PBM spray-coated membranes over commercial PES membranes for use in membrane bioreactors (MBR) for wastewater treatment systems with reduced maintenance over longer operation periods.

## 1. Introduction

Membrane bioreactors (MBRs) have been used for more than two decades to improve the water recovery of industrial and domestic wastewater treatment processes. They combine conventional biological degradation with a physical separation process using a membrane as barrier [1]. Commercially available ultrafiltration (UF) PES membranes are employed in MBRs, due to their high mechanical and chemical stability, to separate the suspended solids from the feed wastewater [2]. Plate and frame modules installed in MBRs consist of many individual flat sheet membranes mounted at a defined distance from each other. The permeate pump is connected to the module to withdraw the effluent through the membranes while rejecting the contaminants in the feed stream [2,3]. Consequently, membrane fouling is an inevitable phenomenon that poses a significant threat to achieving highly efficient filtration in the long run.

Fouling refers to inorganic/organic dissolved and suspended solids attached to the membrane surface and inside the pores. It increases the applied pressure across the membrane (also known as transmembrane pressure (TMP), thus requiring an increase in the pump energy, affecting the flux and leading to the need for membrane cleaning or replacement [4]. For a constant pressure-driven process, fouling results in flux decline, whereas under constant flux conditions, the applied pressure (TMP) increases to maintain the set volume flow. UF membranes are particularly susceptible to fouling phenomena due to the characteristics of wastewater, which contains various contaminants, such as solids, extracellular polymeric substances (EPS), and other macromolecules [5]. The increasing pressure drop over the membrane results in lower permeability which is compensated for by higher TMP values. The possible formation of a fouled cake layer causes an increase in the compaction force across it, thus resulting in a steep increase in TMP known as the ‘sudden TMP jump’ [6]. Progressive internal fouling is known to be irreversible, and in the long term will result in irrevocable permeate flux, leading to costly membrane replacement.

Fouling in constant flux operation is even more severe, since the particulate accumulation does not decrease with time as it does with constant TMP (where the flux decreases over time). The flux or volume flow per unit area of the membrane above which the flux does not increase linearly with increasing TMP is defined as the critical flux [6,7]. Once the critical flux is exceeded, irreversible fouling occurs due to the self-accelerating effect of particle deposition, contributing to the exponential increase in TMP. Beyond the critical point, there is no linear relationship between flux and pressure. The degree of fouling and the membrane type and geometry determines the cleaning activities that must be performed to restore the initial TMP and permeability [8]. Consistent membrane cleaning improves overall system performance but is expected to increase energy consumption and operation cost [9]. Harsh chemical cleaning is rarely carried out and is more relevant in the long-term as a last resort before membrane replacement [10]. Higher scouring in UF membrane modules in submerged MBR systems reduces membrane fouling at the expense of higher energy consumption due to bubble formation with inefficient air compressors. Cross-flow enhancement with the filtration blower is energy-intensive, and varies greatly with air bubble configuration. Feed- or back-pulsing have been applied, and their effectiveness validated in various studies performed with all types of module configurations [9,11]. In general, process optimization techniques to mitigate fouling are aimed at lowering the TMP at a given flux to reduce the specific energy consumption [12]. Nevertheless, prevention of fouling phenomena should be prioritized over cleaning procedures to further reduce consumables and chemicals addition. Some of the methods adopted to improve the fouling properties of UF PES membranes are enzyme-catalyzed modification, addition of macromolecular modifiers, UV treatment, etc., as listed in Table 1.

Most of the existing techniques for the enhancement of PES membranes with impressive anti-fouling properties involve complex processes such as grafting copolymers and manufacturing additives that add to the cost and effort of membrane fabrication. This study focuses on the development of a coating layer that induces low-fouling properties to readily available PES membranes using a simple and facile technique.

One efficient fouling mitigation approach was implemented in a pilot MBR by Deowan et al. [14], and was based on the surface modification of a commercial UF membranes by means of an anti-fouling coating, obtained via polymerization of the novel polymerizable bicontinuous microemulsion (PBM) reported by Galiano et al. [15,16]. The PBM-coated membranes contained the homemade polymerizable surfactant acryloyloxyundecyltriethylammonium bromide (AUTEAB), which showed antimicrobial activity against Gram-positive and Gram-negative bacteria [17]. Investigations by Galiano showed that homemade surfactant AUTEAB was more suitable for PBM composition than commercial surfactants such as dodecyltrimethylammonium bromide (DTAB), because DTAB does not contain an acrylate group, which limits its ability to polymerize or chemically embed itself in the polymer matrix [19]. Another incorporated co-surfactant was 2-hydroxyethylmethacrylate (HEMA), bearing a polar hydroxyl group (–OH) able to confer more hydrophilic properties to the membrane surface [19]. The preparation and polymerization of the microemulsion in a bicontinuous state was confirmed by Galiano et al. via conductivity measurements (showing the need for 30–60% water content) and SEM analyses of the PBM-coated PES membranes, showing the presence of bicontonuous nanostructures [19]. Upon polymerization, the PBM coating forms a solid interconnected polymer matrix with a bicontinuous ‘sponge-like’ structure, with the advantage of being a highly resistant coating with meander-shaped pores [19]. Moreover, the polymerized PBM coating significantly reduces the fouling propensity of commercially available PES membranes, as verified via scanning electron microscopy [20]. Galiano et al. [21], Deowan et al. [20], and Gukelberger et al. [22] have extensively studied the PBM membrane coatings resulting from redox-induced PBM polymerization. Moreover, photo-initiated UV-LED polymerization of PBM was also investigated by Galiano and Schmidt et al. [23], where the curing time of PBM could be reduced to 30 s.

All previous membrane PBM coatings have been obtained using casting knives with varying wet layer thickness. However, spray-coating technology has recently come into focus due to the very high homogeneous coating layer achieved, easy scalability, and high material efficiency [13], even though it has only been applied so far for small-scale membranes in laboratory tests. For example, membrane electrode assemblies (MEA) are a major driver of costs in fuel cells, and contain platinum (Pt) as a catalyst for the redox reaction at the electrodes. The platinum has been sprayed onto the MEA using an ultrasonic nozzle to produce thin coating layers, reducing both material input and investment costs [24]. Sparks et al. [25] and Li et al. [26] fabricated super-hydrophobic surfaces on glass substrates with organic substances using handheld air-brush pistols. The coated area was customized, and the material was further polymerized via UV irradiation [26]. Applications vary, and a specific spray mist and droplet size is required to achieve very thin coating layers. Spray coating technology is based on the principle of atomization, which can be expressed as a high relative velocity between the sprayed material and its surroundings. As a result of the high relative velocity, the liquid is atomized into small droplets that change size and shape with the absolute velocity applied. The total product of all the droplets formed via atomization is the spray mist [27].

In the present study, we have used the PBM composition to prepare enhanced UF-PES membrane performances by coating commercially available PES membrane surfaces with the PBM layer using two different techniques, namely: (i) casting-coating, and (ii) spray-coating. The PBM layer coated using both techniques was polymerized via UV irradiation due to its advantages of shorter curing time and energy efficiency. In addition, UV-initiated polymerization allows for the initiation of the polymerization process to be separated from the coating. This implies that the viscosity of the prepared PBM after addition of the photoinitiator would remain the same until it was exposed to UV light for curing. This is a further advantage over redox-initiated polymerization, wherein the addition of the redox initiators initiates curing immediately, so the viscosity of the prepared PBM increases over time, eventually blocking the spray nozzle for coating. The spray-coated PBM layer on commercially available PES membranes has been verified using ATR-FTIR spectroscopy, and the membrane surface behavior studied via water contact angle (CA) measurement. The thickness of the PBM layer coated on an alumina support has been determined using confocal laser microscopy. Membrane performance has been investigated via measurement of water permeability and conduction of low-fouling tests using 100 mg L^−1^ humic acid (HA) solution as a model foulant for feed. The measurements obtained from a commercially available UF-PES membrane have been used as a reference to compare the performance of the PBM-coated membranes with emphasis on the membranes spray-coated with the PBM layer.

## 2. Materials and Methods

### 2.1. PBM Preparation

The formulation of the PBM materials for application in PES membrane coating was published by Galiano et al. [20]. The chemicals used with ultrapure deionized water (electrical conductivity < 1.6 µS cm^−1^) for preparation of the PBM were methyl methacrylate (MMA) as the monomer, 2-hydroxyethyl methacrylate (HEMA) as the co-surfactant, ethylene glycol dimethacrylate (EGDEMA) as a cross-linker, and acryloyloxyundecyltriethylammonium bromide (AUTEAB) as a laboratory-synthesized surfactant [28]. The photoinitiator 1-hydroxycyclohexyl phenyl ketone (Irgacure 184) was finally added to the microemulsion at a concentration of 1.8 wt% of the total weight of the prepared PBM. All the chemicals used for the experiment, except AUTEAB [29], were purchased from Sigma-Aldrich (now Merck) (Darmstadt, Germany) with a purity of 98–99% (analytical grade). The composition and preparation steps of PBM described by Galiano et al. [20] were carried out in a glovebox at a temperature of 20 ± 5 °C purged with inert nitrogen (N_2_) gas to eliminate the presence of atmospheric oxygen to avoid oxygen inhibition. Pressurized dry nitrogen with a technical purity of 99.8% (DIN EN ISO 14175:N1 [30]) was used. The chemicals were mixed in a glass beaker via mechanical stirring until a clear and transparent microemulsion was obtained.

### 2.2. Commercial Membrane as Reference

The commercially available flat sheet Nadir^®^ UP150 membrane (Mann+Hummel International GmbH, Ludwigsburg, Germany) was used as the reference for water permeability tests. It has a nominal pore size of 0.04 µm and a molecular weight cut-off of 150,000 Daltons [31]. The membrane polymer used in the filtration layer was PES on a mechanical support layer (backing material) of polypropylene (PP). The total membrane thickness of the UF PES membrane was 210–250 µm [32].

The UF membrane was mounted on a glass slab such that its PES layer was exposed as coating substrate for the low-fouling PBM layer. Membrane sheets with an active filtration area of 85 cm^2^ were cut out for water permeability and low-fouling tests. The glycerol filling to protect the pore structure of the commercial PES membrane was removed by soaking the membranes in deionized (DI) water for 3 h before to testing.

### 2.3. Low-Fouling Layer Coating

**Figure 1:**
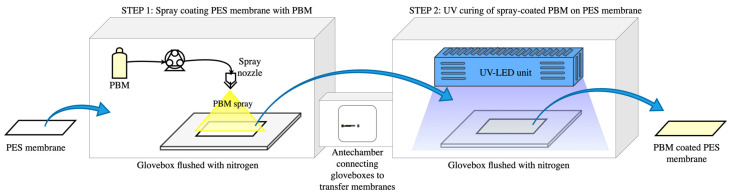
Schematic illustration of the PES membrane spray-coating process with PBM.

The active filtration PES surface of the commercial Nadir^®^ UP150 membrane was used as a substrate to apply the low-fouling PBM layer either using a casting-coating or by spray-coating technique, as described in Section 2.3.1 and Section 2.3.2, respectively. The coated PBM layer was then polymerized via UV irradiation at 300 mW cm^−2^ at a distance of 4 mm. The PBM-coated membranes were exposed to UV irradiation for 1 min to allow polymerization of the PBM layer. Both the coating processes and polymerization of the coated PBM layer were carried out under inert conditions in the glove boxes shown in Figure 1.

Oxygen inhibition generally occurs during the polymerization reaction when carried out under atmospheric conditions. The oxygen molecule (O_2_) reacts with available radicals during the polymer chain growth propagation, and slows down further combination [33]. Therefore, the atmospheric oxygen level inside the glove boxes was kept below 1% wt. by flushing pressurized dry nitrogen (N_2_) gas of technical purity ≥ 99.8%. An oxygen sensor from GHM Messtechnik GmbH (Greisinger Oxy 3690 MP) (Regenstauf, Germany) was installed inside the glove box to monitor the atmospheric oxygen level [34].

#### 2.3.1. Casting-Coating Technique

**Figure 2:**
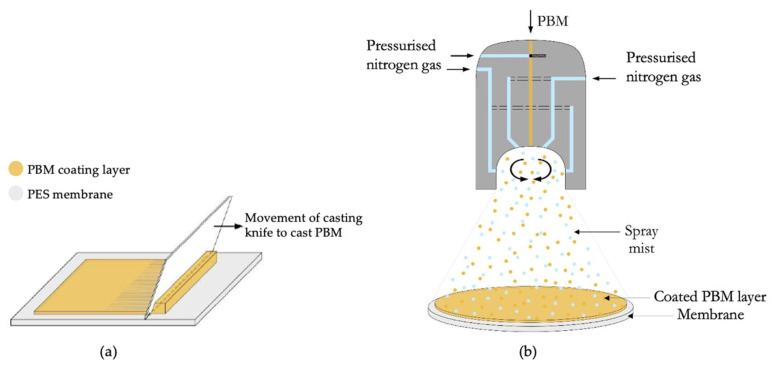
Application of PBM on the PES membrane surface (**a**) via casting-coating and (**b**) via spray-coating.

A spiral casting knife TQC sheen AB3050 (Industrial Physics, Essen, Germany) with a fixed 4 µm wet layer thickness was used to apply the PBM layer on top of the commercially available UF PES membrane [35]. The coating layer was casted manually, as shown in Figure 2a, with a constant casting speed and contact forces for a uniform coating layer (applied pressure).

#### 2.3.2. Spray-Coating Technique

A duel fuel nozzle was used to spray-coat the membranes with PBM, as shown in Figure 2b. It is a pneumatic atomizer in which pressurized (nitrogen) gas assisted the droplet formation from a bulk liquid phase. A peristaltic pump was used to dose the PBM in the nozzle. To achieve the required high relative velocity, a pressurized air duct of high-velocity air surrounds the liquid channel, enabling the atomization. The active liquid was supplied by a separate feed pump to allow independent dosing of the spray parameters [36].

### 2.4. Attenuated Total Reflectance Fourier Transform Infrared Spectroscopy (ATR-FTIR)

The membranes were analyzed using an FTIR spectrometer (Bruker Tensor II) (Ettlingen, Germany) using a platinum ATR module with a diamond crystal-type A225/QHP, a mid-IR source, and deuterated triglycine sulfate (DTGS) detector. The membranes were soaked in DI water (Millipore, electrical conductivity below 1.5 µS cm^−1^) (Merck) (Darmstadt, Germany) to remove the protective glycerol layer and dried at around 60 °C prior to analysis the active filtration PES surface and PBM-coated PES surface in the spectrometer. The membranes fouled with HA were left to dry at room temperature. The fouled membrane surface was then analyzed via FTIR spectroscopy.

### 2.5. Contact Angle Measurement (CAM)

The hydrophilicity of the membrane surface was measured using the sessile drop method in the OCA 15EC setup (DataPhysics Instruments) (Filderstadt, Germany). It was carried out by dispensing a drop of 5 µL ultrapure water from a capillary syringe (diameter 0.72 mm) onto the active filtration surface. The average CA measured three times was reported with the corresponding standard deviations. The membranes were prepared by rinsing three times with DI water to remove the protective glycerol layer followed by drying at 35 °C to remove residual water. The membranes fouled with HA were dried at room temperature and then used for CA measurements.

### 2.6. Scanning Electron Microscopy (SEM)

A scanning electron microscope (Zeiss EVO, MA100, Assing S.p.A, Monterotondo, Italy) was used to examine the cross-section of the membranes and to try to determine the thickness of the coated PBM layer. The membrane morphology was examined at several magnifications—20000×, 4000×, and 15,000× for pristine membranes and 2000×, 4000×, and 10,000× for membranes fouled with HA. Membrane cutouts of about 1 cm^2^ (no pretreatment) were cryogenically cleaved using liquid nitrogen in an attempt to obtain distinct membrane cross-sections. These samples were then sputtered with a thin layer of gold in a 4 min cycle in a sputtering machine (Quorum Q150 RS) (Quorum Technologies Ltd., East Sussex, UK) to prepare them for SEM analysis.

### 2.7. Confocal Microscopy

The laser scanning confocal microscope (Lext OLS4100) (Evident Europe GmbH, Hamburg, Germany) was used to measure the thickness of the coated PBM layer on two substrates: the PES membrane surface (no pretreatment) and alumina [37]. The multi-layer film thickness mode was used with 405 nm laser beam to determine the thickness of the coated PBM layer.

### 2.8. Membrane Cross-Flow Test Unit

A laboratory cross-flow test unit (SIMA-tec LSta05) (SIMA-tec, Schwalmtal, Germany) was used to evaluate performance of the UF membranes [38]. The flow was measured using a magnetic inductive flowmeter (ABB FEX300) (ABB Measurement and Analytics, (Goettingen, Germany), and the TMP was measured using absolute pressure sensors (WIKA Type A-10) (WIKA, Klingenberg, Germany). Both the permeability test (with DI water) and low-fouling tests (with HA solution) were performed at a constant cross-flow velocity (CFV) of 28 L h^−1^ across a membrane sample of 84 cm^2^ active filtration area. The cross-flow, permeate flux and TMP were continuously monitored by the SIMA-tec control unit (sampling time of 60 s), which also reported the water permeability of the membrane samples calculated via the following relation:

(1) $$Permeability=\frac{Flux}{TMP}$$

where flux is the permeate flow rate per unit membrane area expressed in L m^−2^ h^−1^, and TMP is expressed in bars. The pure water permeability (PWP) test was carried out at a constant TMP of 300 mbar with DI water (electrical conductivity ≤ 50 µS cm^−1^). A typical model foulant—HA purchased from Alfa Aesar (Thermo Fisher Scientific, Kandel, Germany) (CAS: 1415-93-6) was used to prepare 100 mg L^−1^ feed solution for the membrane low-fouling test in the cross-flow test unit. The feed solution and permeates in the study were analyzed for TOC using the analyzer TOC-L CPH/CPN (Shimadzu, Darmstadt, Germany).

### 2.9. Critical Flux Determination

The low-fouling test protocol was performed in the laboratory cross-flow unit according to the standard flux-step method developed by Clech and Jefferson et al. [39]. This protocol was suitable for conducting short-term tests and collecting useful data for a comparative study of the fouling propensity of different membranes. The flux-step method involved changing the flux by a step height of 12 L m^−2^ h^−1^ (approximated from 11.8 L m^−2^ h^−1^) after a time interval of every 30 min (see Section 3.2.2). The flux was kept constant during the fixed time interval of 30 min (chosen on the basis of the time required to obtain a stable flux-step in the cross-flow unit). The flux was increased in steps until one of the following cross-flow unit thresholds was reached: (i) a maximum rate of increase of TMP at each flux-step (dP/dt) of 10 mbar min^−1^, or (ii) a maximum operating TMP of 6 bars. The ascending phase of the flux-step protocol was followed by the descending phase, in which the flux was reduced in steps of 12 L m^−2^ h^−1^ until the initial flux value at the start of the test was reached. The TMP was continuously monitored throughout the test protocol and reported for performance comparison. The flux step of 30 min, at which the slope of increase in TMP was measured to be more than 0.1 bar (dP/dt ≥ 3 mbar min^−1^) in the ascending phase indicated the onset of critical fouling in this study. The TMP was held stable below the threshold of 3 mbar min^−1^ for each flux step, indicating a linear increase in TMP with permeate flux. The critical fouling of the membranes was determined by comparing the TMP at the same flux levels during the ascending and descending phases.

## 3. Results and Discussion

### 3.1. Membrane Morphology and Properties

#### 3.1.1. ATR-FTIR Spectroscopy

**Figure 3:**
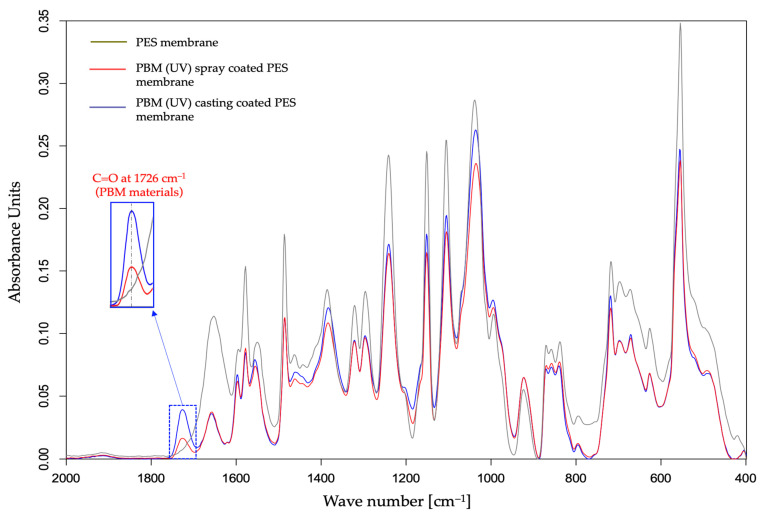
Detection of a PBM coating layer using the ATR-FTIR spectral curve of a UF PES membrane surface.

The characterization of the PBM coating on UF PES membrane was carried out by superimposing the ATR-FTIR spectral curves of the pristine PES membrane and the PBM-coated membrane surfaces (Figure 3).

The absorption peak of C=O double bond at around 1726 cm^−1^ in the ATR-FTIR spectra is a marker of the presence of the PBM-coated layer on the PES membrane surface [20]. In fact, all the components present in PBM contain the carbonyl functional group (C=O) which is not present in a commercial PES membrane surface. Interestingly, the amplitude of the absorption peak of C=O group shown in Figure 3 was greater for the membrane surface casting-coated with PBM than for spray-coated one. This indicates a thicker casting-coating layer than the spray-coated layer. Thus, ATR-FTIR spectroscopy provided confirmation on the presence of the PBM coating layer on the PES membrane surface with a qualitative assessment of the coated PBM layer.

#### 3.1.2. SEM Imaging

**Figure 4:**
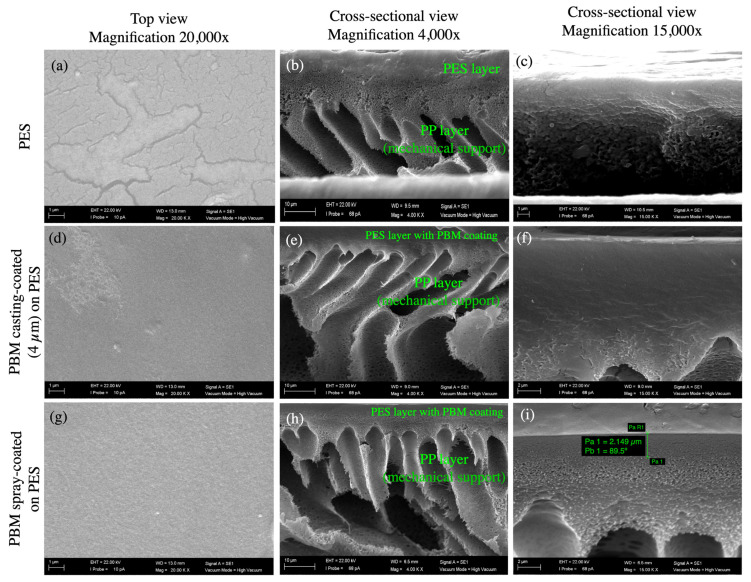
SEM micrographs of the top and cross-sectional views of (**a**–**c**) the commercial PES membrane surface; (**d**–**f**) PBM casting-coated on the PES membrane surface; and (**g**–**i**) PBM spray-coated on the PES membrane surface obtained at magnifications of 20,000× (**left** column); 4000× (**middle** column); and 15,000× (**right** column). (**i**) The thickness of the spray-coated PBM layer was estimated to be about 2.15 µm, as measured using the SEM micrograph (highlighted in green bars).

The typical porous structure of the PES membrane was visible in the top view SEM micrograph (Figure 4a). However, these porous structures were not visible at the same magnification when the PBM coating was applied on the PES membrane surface (Figure 4d,g), indicating a denser structure on top of the PES membrane. The cross-sectional images of the membrane samples clearly showed the PES layer on top of the porous mechanical PP support structure (Figure 4b,e,h). However, the coated PBM layer could not be clearly distinguished from the PES surface in the cross-sectional view (Figure 4c,f,i), probably due to its very low thickness and good adhesion to the PES surface. Therefore, the thickness of the coated PBM layer was estimated using an alternate technique: confocal microscopy.

#### 3.1.3. Determination of Thickness of Coated PBM Layer Using Confocal Microscope

The PBM layer was coated using identical methods under inert conditions (nitrogen atmosphere) on two different substrates: the PES membrane surface and alumina. The thickness of the casting-coated PBM layer on the PES membrane using a 4 µm casting knife was measured to be approximately 4.85 ± 1.00 µm. However, the thickness of the spray-coated PBM layer on the PES membrane surface could not be measured via the same technique due to the deposition of a very thin layer (estimated to be less than 2.5 µm) that could not be easily distinguished from the PES membrane surface using the confocal microscope.

Alternatively, the thickness of the casting-coated PBM layer on the alumina substrate was measured to be 4.6 ± 0.5 µm, a reasonable 5% difference from the PES membrane surface. Thus, the estimated thickness of the spray-coated PBM layer on alumina substrate was found to be 1.7 ± 0.1 µm, as summarized in Table 2. The lower deviation in the thickness measurement of the spray-coated PBM layer was attributed to the automated spray-coating technique, which produces a relatively more homogeneous coating layer than the manual casting-coating method.

#### 3.1.4. Water Contact Angle (CA)

The membrane samples were wetted and then dried to measure the CA via the sessile drop method, as shown in Table 3. The improvement in the hydrophilicity of the PBM-coated membranes was indicated by the reduction in the CA. It is known from the literature that increased hydrophilicity reduces fouling, as it is mainly caused by more hydrophobic solutes such as natural organic matter (NOM) present in MBRs. NOMs can be easily adsorbed and deposited on membranes with a more hydrophobic character [40].

### 3.2. Water Permeability and Rejection Tests

The performance of a membrane is determined by its permeability (L m^−2^ h^−1^ bar^−1^) at constant flux (L m^−2^ h^−1^). For example, pure water permeability (PWP) is a useful metric for selecting a suitable membrane for system implementation; the lower the PWP, the larger the membrane area required to achieve the capacity demanded by a system will be. Guzelot et al. computer-simulated the water permeability of PBM dip-coated UF hollow fiber membranes at 25 °C as a function of coating thickness [41]. The simulated permeability shows a dramatic decrease in water permeability above a coating thickness of 10 µm, wherein the water permeability drops below 30 L m^−2^ h^−1^ bar^−1^. To achieve water permeability above 50 L m^−2^ h^−1^ bar^−1^, the coating thickness should be less than 5 µm [41].

#### 3.2.1. Pure Water Permeability (PWP)

**Figure 5:**
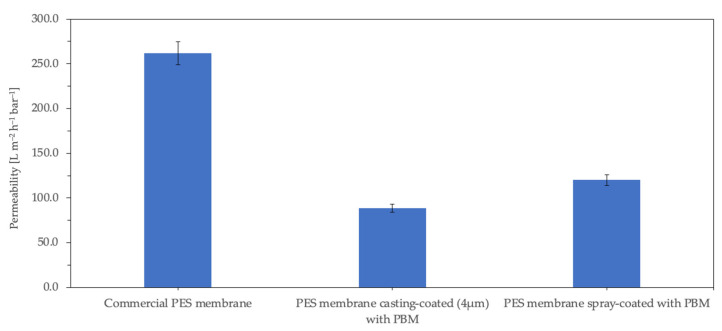
Permeability of the membrane samples measured with DI water (electrical conductivity ≤ 50 µS cm^−1^).

The PWP of the commercial PES membrane and PBM casting-coated and spray-coated membranes was measured for 12 h using DI water in a cross-flow unit. The measured PWP of the commercial PES membrane was 262 ± 4.0 L m^−2^ h^−1^ bar^−1^ (rated by the manufacturer to be ≥285 [31]). Figure 5 shows that the PWP of the PBM casting-coated membrane decreased by 66%, while that of the PBM spray-coated membrane decreased by 54% compared to the uncoated commercial PES membrane. Galiano reported the pore size distribution of the PBM-coated membranes (using a 250 µm casting knife) to be 25% lower than that of the PES membranes [19]. This reduction in PWP was attributed to the coating of PBM on the PES membrane surface; the thicker the coating, the greater the surface resistance of the membrane, and therefore the greater the reduction in PWP. Thus, the thinner spray-coated PBM layer resulted in the PES membrane having a 12% lesser PWP-drop than the thicker casting-coated PBM layer membrane (see Figure 5).

The PWP of membranes for use in MBRs should be sufficiently high, as the membrane modules are typically operated by low-pressure suction pumps, and the maximum suction pressure usually recommended by the manufacturers is below 400 mbar [32]. Therefore, to achieve a typical water flux of approximately 20–25 L m^−2^ h^−1^ in MBR, at operating suction pressures of 250–350 mbar, a minimum water permeability of 60–100 L m^−2^ h^−1^ bar^−1^ is required for the membranes. The PWP of both the PBM 4µm casting-coated membrane and the PBM spray-coated membrane were measured to be 89 ± 0.5 L m^−2^ h^−1^.bar^−1^ and 120 ± 5.5 L m^−2^ h^−1^ bar^−1^, respectively, well within the desired operating range for MBR applications. However, it should be noted that the PWP does not significantly drop when submerged in the activated sludge of MBRs.

#### 3.2.2. Low-Fouling Tests

**Figure 6:**
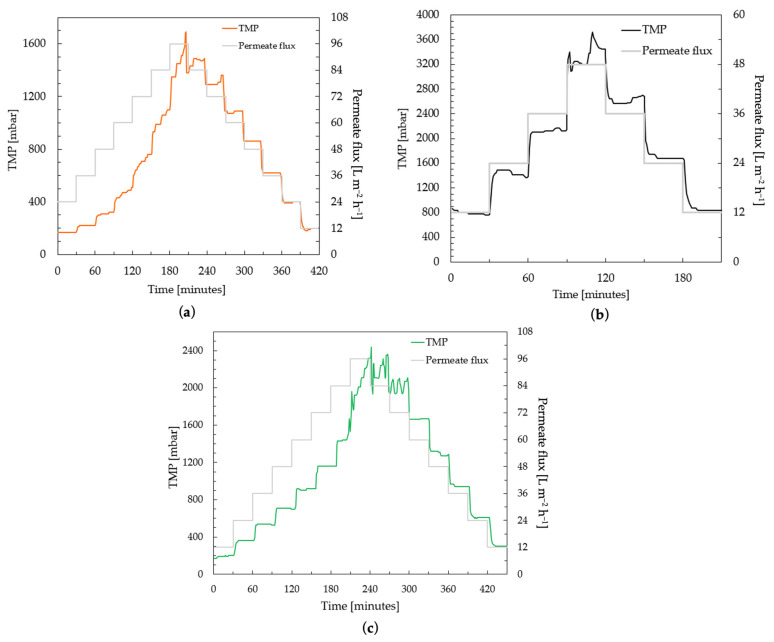
Determination of critical flux by flux-step test protocol using 100 mg L^−1^ HA solution of (**a**) commercial PES membranes; (**b**) a PES membrane casting-coated (4 µm) with PBM; and (**c**) a PES membrane spray-coated with PBM.

The performance and long-term behavior of the membranes were investigated by a critical flux test carried out according to the protocol given in Section 2.8. The critical flux test helps to determine the sustainable conditions of the membranes studied while maintaining an acceptable performance. Figure 6 shows the flux-step profile of three different PES membrane samples compared in this study.

**Figure 7:**
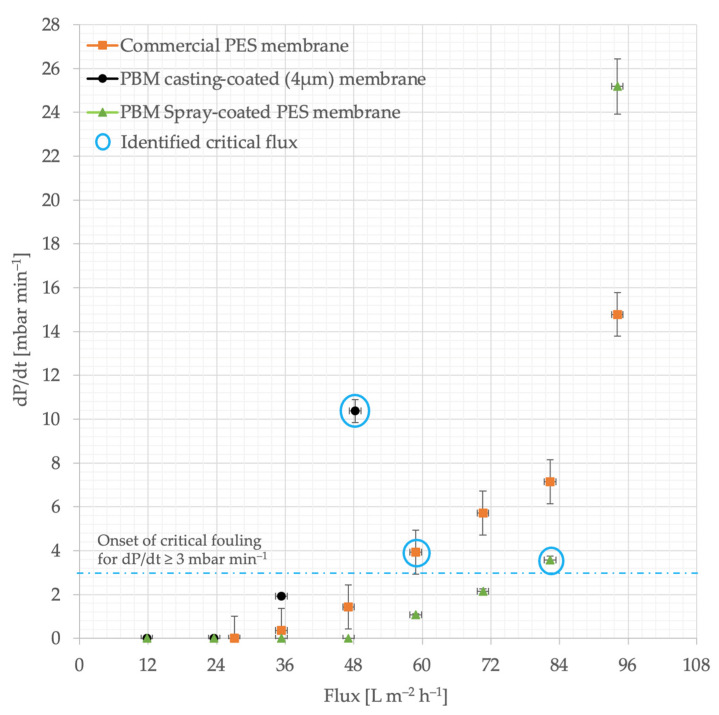
Identifying the onset of critical fouling using the rate of TMP increase (dP/dt) versus flux. The precise height of the flux step was 11.8 L m^−2^ h^−1^, which was approximated to 12 L m^−2^ h^−1^.

The commercial PES membrane shows smooth performance, i.e., the increase in TMP was linear to the increase in permeate flux steps, up to 48 L m^−2^ h^−1^ in the ascending phase (Figure 6a). The TMP jump continues to increase at fluxes higher than 48 L m^−2^ h^−1^. When the flux is reduced to from 60–48 L m^−2^ h^−1^, the TMP shows a stable level again. Similar smooth behavior was observed for the PBM casting-coated membrane up to 36 L m^−2^ h^−1^, and for the PBM spray-coated membrane up to 72 L m^−2^ h^−1^ (Figure 6b and Figure 6c, respectively). The rate of increase in TMP was observed to be significant (dP/dt ≥ 3 mbar min^−1^) in the commercial PES membrane for flux steps above 48 L m^−2^ h^−1^. This flux (at which the rate of change in TMP (dP/dt) exceeded the threshold of 3 mbar min^−1^) can be considered the critical flux, which indicated the onset of the critical fouling of the membrane (Figure 7). All the membranes were inferred to be critically fouled due to (i) an increasing fouling rate at every flux step, indicated by rising dP/dt; and (ii) the higher TMP observed at a flux step in the descending phase, as compared to that in the ascending phase [39].

The critical fluxes of the commercial PES membrane, PBM casting-coated and spray-coated membranes were identified at 60 L m^−2^ h^−1^, 48 L m^−2^ h^−1^ and 84 L m^−2^ h^−1^, respectively due to the absence of a smooth behavior (stable TMP). This represents a 40% improvement in performance of the PBM spray-coated PES membrane over the commercial PES membrane. The TMP returns to smooth behavior in the commercial PES membrane, PBM casting-coated and spray-coated membranes after 60 L m^−2^ h^−1^, 36 L m^−2^ h^−1^ and 60 L m^−2^ h^−1^, respectively, in the descending phase.

The casting-coated membrane generally has higher TMP than the spray-coated and uncoated membrane at the same flux levels. This is due to the greater thickness of the PBM coating. The added resistance of the PBM coating on the PES membrane surface also results in better flux control, as indicated by the stable TMP values of 12–72 L m^−2^ h^−1^ for the PBM spray-coated membranes and 12–36 L m^−2^ h^−1^ for the PBM casting-coated membranes in the descending phase (Figure 6b,c). The flux-step test shows that the trade-off for effective low-fouling is the thickness of the low-fouling coating.

#### 3.2.3. Rejection Rate

The 100 mg/L HA solution feed (pH = 8.0 ± 0.2) was analyzed to contain TOC of 46 mg/L ± 6.50%. The feed was filtered during the low-fouling tests to produce permeate with lower TOC levels as shown in Table 4. The PBM casting-coated membrane has 5% points higher rejection rate than the PBM spray-coated membrane which can be attributed to its thicker coating.

In 2013, Galiano and Deowan found the molecular weight cut-off (MWCO) of the PES membrane to be 270 kDa (commercially reported to be 150 kDa), and that of the PBM casting-coated PES membrane to be 100 kDa, as measured using a gel permeation chromatography technique [19,31,42]. This 64% reduction in the MWCO of the PBM coating reported by Galiano and Deowan suggests that the PBM coating layer is more effective in rejecting the larger humic acid molecules, which typically lie within a range of 2–1500 kDa [43].

### 3.3. Characterisation of the Membranes Fouled with HA

**Figure 8:**
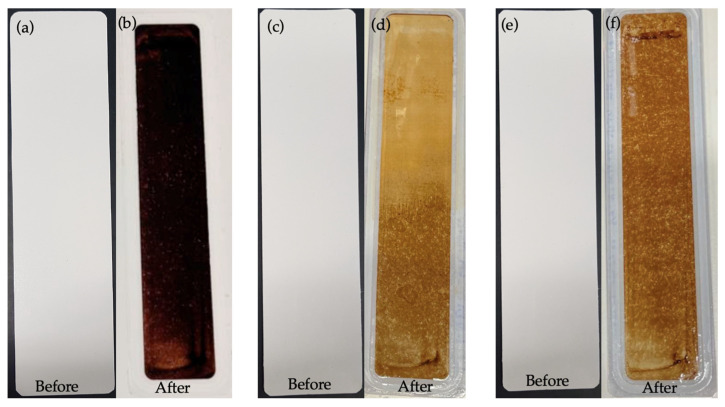
Appearance of membranes before and after low-fouling tests using 100 mg L^−1^ HA solution: (**a**,**b**) commercial PES membranes; (**c**,**d**) a PES membrane casting-coated (4 µm) with PBM; and (**e**,**f**) a PES membrane spray-coated with PBM.

The fouling layer of HA on the membrane samples was visibly indicated by a darker brownish shade for a thicker fouling layer and an ochre-yellowish shade for a lighter fouling layer, as shown in Figure 8b,d,e. It could be inferred from the visual impressions of the fouled membranes that a slightly lighter fouling layer deposited on the PBM-coated membranes than on the commercial PES membrane, showing their low-fouling property.

#### 3.3.1. ATR-FTIR Spectroscopy of PES Membranes Fouled with HA

**Figure 9:**
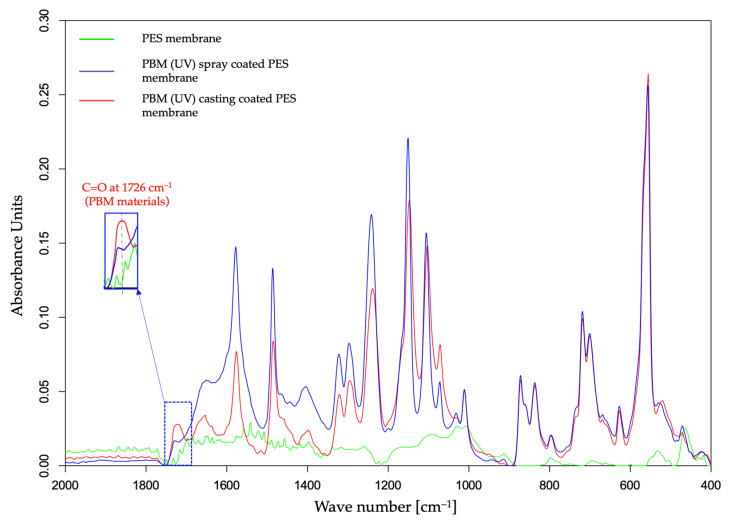
ATR-FTIR spectral curve of UF PES and PBM-coated PES membrane surfaces fouled with HA.

The deposition of a thicker fouling layer on the PES membrane surface was indicated by the attenuated absorbance amplitude in the ATR-FTIR spectral curve (Figure 9). Most of the absorbance peaks for the PBM-coated membranes were detected similar to their pristine state, but with lowered amplitudes due to the HA fouling layer (Figure 3 and Figure 9). The thicker the fouling layer, the lower was the absorbance amplitude on the ATR-FTIR spectral curve. Thus, the PBM spray-coated and casting-coated membranes showed deposition of a thinner fouling layer than the PES membrane. Further, the presence of PBM coating after the fouling tests was indicated by the absorbance peak of C=O at 1726 cm^−1^.

#### 3.3.2. SEM Images of PES Membranes Fouled with HA

**Figure 10:**
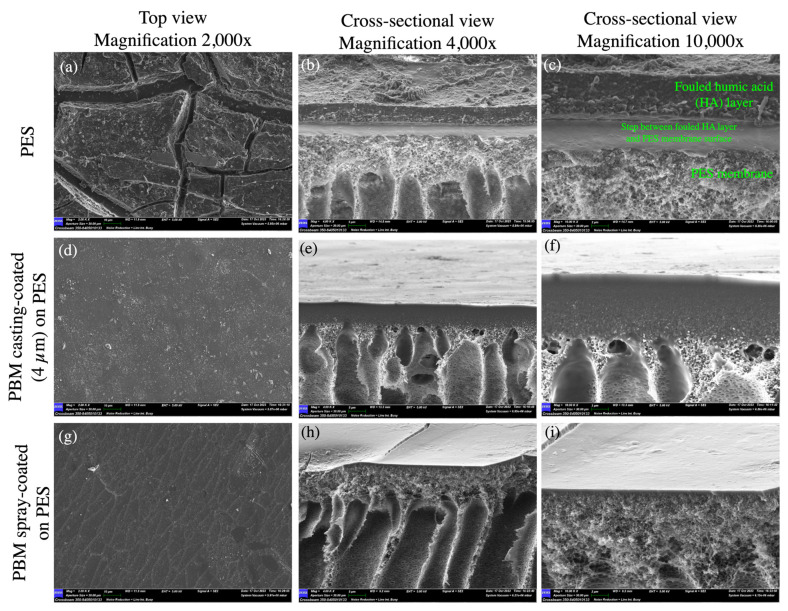
SEM micrographs of the top and cross-sectional views of (**a**–**c**) the commercial PES membrane surface; (**d**–**f**) PBM casting-coated on the PES membrane surface; and (**g**–**i**) PBM spray-coated on the PES membrane surface fouled with humic acid after the step-test protocol.

**Figure 11:**
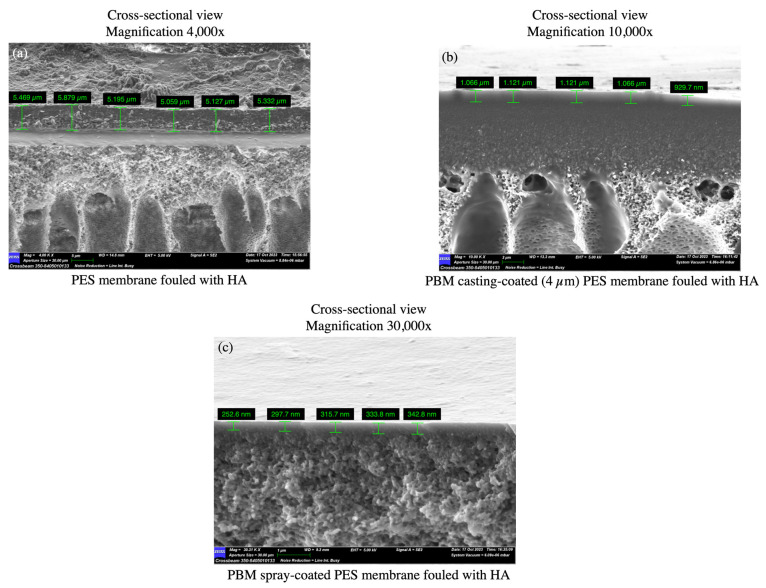
SEM micrographs of cross-sectional views used to estimate the thickness of the fouled HA layer deposited on (**a**) the commercial PES membrane surface; (**b**) PBM casting-coated on the PES membrane surface; and (**c**) PBM spray-coated on the PES membrane surface after the flux-step test protocol.

The top-down SEM images of the fouled membranes show the deposited HA layer with cracks, which appeared after the drying of the fouled membranes at room temperature (Figure 10a,d,g). The dried thick HA layer can be seen as a step on the PES membrane surface in the SEM micrograph (Figure 10c). The thick HA layer was more distinctly visible as a cake layer on the PES membrane (5.0–6.0 µm) as compared to the thinner fouled layers on the PBM casting-coated membrane (0.9–1.2 µm) and PBM spray-coated membrane (0.25–0.35 µm) (Figure 11a–c). This further exhibits the low-fouling nature of the PBM-coated membranes.

#### 3.3.3. CAM of PES Membranes Fouled with HA

HA is known to be a complex mixture comprising both hydrophilic and hydrophobic compounds such as fulvic acid and humins, respectively [44]. Thus, the hydrophilicity of a polymer surface can be affected by the thickness of the deposited HA layer. In 2012, Platkowska-Siwiec and Bodzek showed that the tendency for hydrophobic membranes to be fouled is higher than that of hydrophilic membranes; they ascribed this tendency to the adsorption of the negatively charged functional groups of NOM on the positively charged PES polymer [45]. Such a trend was observed via the greater decrease in the CA of the HA fouled layer on the comparatively hydrophilic PBM-coated membrane than on the PES membrane (Table 5).

The increase in the CA of the HA-fouled PES membrane surface was attributed to the deposition of a thicker fouling layer that created a barrier, hindering water molecules from interacting with the PES membrane surface (Figure 10a–c).

## 4. Conclusions

This work is focused on reducing the fouling propensity of commercial UF PES membranes for membrane bioreactor (MBR) applications through a coating based on a polymerizable bicontinuous microemulsion (PBM). The commercial PES membrane surface was modified either via casting or spray-coating techniques. The presence of the PBM coating was successfully detected using ATR-FTIR spectra. The thickness of the spray-coated PBM layer on alumina substrate was measured with a laser confocal microscope to be around 60% thinner than the casting-coated layer. The advantage of depositing a thinner spray-coated PBM layer over a casting-coated layer was demonstrated by a 12% improvement in PWP-drop and a 60% improvement in critical flux (obtained from a fouling flux-step test with HA as the model foulant), with agreeable trade-offs in hydrophilicity (1.4% lower) and rejection rate (5% lower). Moreover, the critical flux of the membranes spray-coated with PBM was 40% higher than that of the commercial PES membrane. The low-fouling propensity of the spray-coated PBM layer was exhibited by the characteristics of the fouled HA layer, which showed that a thinner fouling layer deposited on the PBM spray-coated membrane as compared to the other membranes.

In contrast to classical casting-coating, spray-coating also offers great advantages in terms of system flexibility, scalability of the coated surface area, controlled layer thickness and low membrane sheerness. Finally, based on the promising results, spray-coating technique with PBM materials will be further studied, especially in the harsh environment of MBRs.

## 5. Patents

The compositions of the PBM materials used in this work were submitted for a European patent published in 2016 and granted in 2019 [15].

## Figures and Tables

**Table 1:** Some ameliorated UF PES membranes with anti-fouling properties in the literature.

Membrane Modification Approach	Improved Membrane Performance	Ref.
Addition of polyvinylpyrrolidone (PVP), polyethylene glycol (PEG) and (Pluronic^®^, Plu) to the PES membrane prepared via a non-solvent-induced phase separation (NIPS) method.	Compaction study (450 kPa for 2 h) showed PES membrane had highest pure water flux (PWF) (~120 L m^−2^ h^−1^) followed by PES-Plu (~70 L m^−2^ h^−1^), PES-PEG (~55 L m^−2^ h^−1^), and PES-PVP (~40 L m^−2^ h^−1^). UF experiments of the anti-fouling membranes with 0.1 g L^−1^ bovine serum albumin (BSA) at 300 kPa showed that PES-Plu had the highest permeate flux and highest protein rejection rate.	[13]
Binding titanium dioxide (TiO_2_) nanoparticles using dopamine (DA) adhesive on PES membrane surface via dip-coating.	Hybrid DA-TiO_2_ PES membrane showed pure water flux (PWF) improvement from 79.9 L m^−2^ h^−1^ to 962 L m^−2^ h^−1^. A fouling test with 1 g L^−1^ BSA at 0.1 MPa showed rejection improvement in rejection rate of 10–20%.	[14]
Graft polymeric oligomers using ferulic acid modifier and laccase bio-catalyst on the PES membrane.	The PWF of modified membranes reduced by an average of 10%. Antifouling property was improved due to 94% reduction in protein adsorption (BSA).	[15]
Cast membranes using PES/PEG blend and graft PEG methacrylate on PES (PES-g-polyPEGMA) via an NIPS method.	The PWF of PES-g-polyPEGMA was higher (~170 L m^−2^ h^−1^) than the PES/PEG membrane. Fouling tests with BSA (1 g L^−1^) at 0.1 MPa and surface velocity of 2 kg h^−1^ showed higher rejection rate (~90%) than PES-g-PEGMA membrane (~98%). The latter was highlighted due to its improved anti-fouling properties.	[16]
Cast PES membranes using nanocomposite graft copolymer additive—(poly(maleic anhydride-co-glycerol), PMG) nanoparticles via an NIPS method	PMG-PES membranes had highest PWF (~908 L m^−2^ h^−1^) than PES membranes (~150 L m^−2^ h^−1^). Fouling tests using BSA at 1 bar showed a 98% rejection rate for the PMG-PES membrane as compared to 69% for the PES membranes.	[17]
Cast composite membrane using molybdenum disulfide-iron oxyhydroxide, MoS_2_-FeOOH/PES via a phase inversion method.	PWF of MoS_2_-FeOOH/PES membranes was 1.7 times higher (~385.3 L m^−2^ h^−1^) than PES membranes (~225 L m^−2^ h^−1^). Fouling tests using BSA (0.5 g L^−1^) at 0.1 MPa showed a rejection rate of 91% for the anti-fouling composite membranes and 96% for the PES membranes.	[18]

**Table 2:** Thickness of coated PBM as estimated via confocal microscopy.

Coating Substrate	PBM Coating Technique	Thickness of Coated PBM Layer
PES membrane surface	Casting-coated	4.85 ± 1.00 µm
PES membrane surface	Spray-coated	Could not be determined ^1^
Alumina surface	Casting-coated	4.60 ± 0.50 µm
Alumina surface	Spray-coated	1.70 ± 0.10 µm

^1^ Deviation for the measurement was more than 40% and hence not accurate.

**Table 3:** Hydrophilicity of the PES membranes coated with PBM based on CAM.

Membrane Surface	CAM (Average)	Reduction in CAM
PES	73° ± 3°	-
Casting-coated PBM on PES	65° ± 3°	Reduced by 11.0% from PES
Spray-coated PBM on PES	66° ± 2°	Reduced by 9.6% from PES

**Table 4:** Rejection of total organic carbon measured by filtering 100 mg L^−1^ HA solution.

Membrane Sample	Rejection Rate	Improvement in Rejection Rate
Commercial PES	33%	-
PES casting-coated with PBM	55%	Increased by 22% from PES
PES spray-coated with PBM	50%	Increased by 17% from PES

**Table 5:** CAM of the PES and PBM-coated PES membranes fouled with HA.

Membrane Surface	CAM (Average)	Change in CA after HA Fouling
PES	86° ± 1°	Increased
Casting-coated PBM on PES	57° ± 1°	Decreased
Spray-coated PBM on PES	50° ± 2°	Decreased

## Data Availability

The data presented in this study are available on request from the corresponding authors.
